# Comparing the combination therapy of ezetimibe and atorvastatin with atorvastatin monotherapy for regulating blood lipids: a systematic review and meta-analyse

**DOI:** 10.1186/s12944-018-0880-8

**Published:** 2018-10-17

**Authors:** Cong Ai, Shanshan Zhang, Qiao He, Jingpu Shi

**Affiliations:** 1 The Department of Clinical Epidemiology, Institute of Cardiovascular Diseases and Center of Evidence Based Medicine, The First Affiliated Hospital, China Medical University, No.155 Nanjing Bei Street, Heping District, Shenyang, 110001 Liaoning China; 2 The Institution of Translational Medicine, China Medical University, No.77 Puhe Road, Shenyang North New Area, Shenyang, 110122 Liaoning China

## Abstract

**Background:**

Although there were many studies reporting the combination therapy of Ezetimibe and Atorvastatin’s efficacy and Atorvastatin monotherapy’s, the conclusions were controversial. Therefore, a systematic review and meta analysis of combination therapy and monotherapy were conducted.

**Methods:**

PubMed, Cochrane Library and Embase were searched for studies of the combination therapy of Ezetimibe and Atorvastatin and Atorvastatin monotherapy published up to October 20, 2017. Two investigators assessed the articles for eligibility and evaluated quality.The changed values and the efficacy of low-density lipoprotein cholesterol (LDL-C), high-density lipoprotein cholesterol (HDL-C), Total Cholesterol (TC) and Triglyceride (TG) indicators were the outcomes. Four doses of the comparisons were included: the combination therapy of Ezetimibe (10 mg) and Atorvastatin (10 mg) (E10 + A10) versus Atorvastatin (20 mg) monotherapy (A20); E10 + A10 vs. A10; E10 + A20 vs. A40; E10 + A40 vs. A80. Review manager software 5.1 was used for quality assessment and Stata version 12.0 software was used for statistical analysis.

**Results:**

eventeen studies (11 publications) were included in the meta analysis. Compared with Atorvastatin monotherapy, the overall efficacy of combination therapy of Ezetimibe and Atorvastatin on lowering LDL-C (MD = − 15.38, 95% CI: -16.17 to − 14.60; I^2^ = 26.2%, *n* = 17), TC (MD = − 9.51, 95% CI: -10.28 to − 8.74; I^2^ = 33.7%, n = 17) and TG (MD = − 6.42, 95% CI: -7.78 to − 5.06; I^2^ = 0%, *n* = 15) and raising HDL-C (MD = 0.95, 95% CI: 0.34 to 1.57; I^2^ = 0%, *n* = 17) was significant. The efficacy of the comparison on HDL-C was largely significant for the different doses.

**Conclusions:**

The overall efficacy and subgroup’s efficacy of combination therapy of Ezetimibe and Atorvastatin on lowering LDL-C, TC and TG was significantly better than Atorvastatin monotherapy’s. The overall and the E10 + A10/A20 group’s effectiveness of combination therapy on rasing HDL-C were significantly.

## Background

The consequences of atherosclerotic disease in the carotid arteries and coronary arteries are serious in human. Patients with atherosclerotic disease are at high risk of cardiovascular and cerebrovascular diseases [1]. Large primary and secondary prevention studies of statins have shown conclusively that lowering low-density lipoprotein cholesterol (LDL-C), Total Cholesterol (TC) and Triglyceride (TG) levels or raising high-density lipoprotein cholesterol (HDL-C) levels reduces cardiovascular events [2, 3]. However, in clinical practice, the treatment of reducing lipid is based on statin monotherapy [4].

Ezetimibe is one kind of lipid-lowering drugs known as cholesterol absorption inhibitors which has different metabolic pathways with statins [5]. Ezetimibe was used in conjunction with many drugs, such as Atorvastatin, Simvastatin, Fenofibric acid, et al. [6, 7]. The comparison has been reported in many studies between combination therapy and statin monotherapy, but the index of the blood lipid level varies considerably [8, 9]. Although there was a systematic review [10] about combination therapy and monotherapy, it was qualitative systematic review without quantitative analysis. Therefore, the purpose of our study was to compare the combination therapy of Ezetimibe and Atorvastatin (E + A) with Atorvastatin monotherapy (A) for regulating blood lipids in the clinical application dose, and summarize the results of comparisons. Subgroup analysis was used to explore whether different doses had impact on the comparison between combination therapy and monotherapy.

## Materials and methods

### Search strategies

The meta-analysis was performed, according to the Preferred Reporting Items for Systematic Reviews and Meta-analysis (PRISMA) statement [11]. Relevant studies were searched from the database of PubMed, Cochrane Library and Embase from inception through October 2017. The search terms included: (Atorvastatin [All Fields] AND Ezetimibe [All Fields] AND combination therapy [All Fields]). We also identified additional references by manually searching for publications that were cited in the included articles and related reviews.

### Study selection criteria

Inclusion criteria are as follows: (1)Study design was randomized controlled trials. (2)All participants were 18 to 90 years old. (3)All participants had minimum treatment duration of 4 weeks. (4)Full text publication is available. (5)The results of the study should include changes of LDL-C, HDL-C, TC and TG.

Exclusion criteria: (1)Reviews, animal studies, case reports, and personal experience summaries. (2)Only the latest paper was included into our final analysis related to duplicated studies and reports. (3)Original data displayed as figures or no original data reported.

### Data extraction

Data extraction was conducted by two investigators independently. On the basis of the inclusion criteria, the following information was collected: first author’s name, date of publication, country, study design, number of patients in each group, outcome of the indicators. If the two investigators had disagreements during data extraction, the third investigator was invited to assess the articles through discussion.

### Quality assessment and intervention description

Quality assessment: The risk of bias in the included studies was assessed by the criteria described in the Cochrane Handbook through the tool of Review manager software 5.1 [12]. Each study was assigned a value of “high” “low” or “unclear” according to the following items: random sequence generation (Selection bias), allocation concealment (Selection bias), blinding of participants, personnel (Performance bias) and outcome assessment (Detection bias), incomplete outcome data (Attrition bias), selective reporting (Reporting bias) and other bias [13]. If the two investigators had disagreements during quality assessment, the contradiction was resolved through discussion.

Intervention description: Randomized controlled clinical trial design was adopted in the included studies. The dosage groups in the literature included the combination therapy of Ezetimibe (10 mg) and Atorvastatin (10 mg) (E10 + A10) versus Atorvastatin (20 mg) monotherapy (A20), E10 + A10 vs. A10, E10 + A20 vs. A40 and E10 + A40 vs. A80. (Table 1).

**Table 1:** Characteristics of included studies

Author, year, country, Reference	Study design	Dose (mg)	Included number		LDL-C		HDL-C		TC		TG	
			E + A	A	Mean	Treatment difference (95% CI)	Mean	Treatment difference (95% CI)	Mean	Treatment difference (95% CI)	Mean	Treatment difference (95% CI)
Matsue,Y., 2013,Japan,19	randomized, open-labeled, parallel-group	E10 + A10/A20	115	128	−16.7	(−18.34,-15.06)	0.47	(−2.05,2.99)	−9.6	(−11.07,-8.13)	−5.3	(− 9.59,-1.01)
Teramoto,T., 2012,Japan,20	multicenter, randomized, open-label, parallel-group	E10 + A10/A20	47	46	−10.6	(−15.4,-5.8)	4	(−0.3,8.3)	− 7.6	(− 11.4,− 3.8)	-3	(−18.8,12.7)
Ben-Yehuda,O., 2011,USA,21	multicenter, randomized, double-blind, parallel-arm	(a)E10 + A10/A20 (b)E10 + A10/A20	404 111	408 107	−13.6 − 14.5	(− 16,-11.2) (− 19.1,-9.8)	1.3 4.2	(− 0.5,3.1) (0.7,7.6)	−7.8 − 8.4	(−9.4,-6.2) (− 11.5,-5.3)	− 5.7 − 7.6	(− 8.9,-2.4) (− 13.3,-2)
Zieve,F., 2010,Russia,22	multicenter, randomized, doubleblind, parallel-arm	E10 + A10/A20	515	515	− 14	(− 16,-12)	2	(0.3,4)	−8	(− 9,-7)	−6	(− 9,-3)
Stein,E., 2004,Spain,23	multicenter, randomized, double-blind, active-controlled	E10 + A10/A20	293	303	−14.8	(− 16.74,-12.86)	0.8	(− 0.86,2.46)	− 11.2	(− 12.86,-9.54)	−5.4	(− 10.11,-0.69)
Padhy,B.M., 2013,India,24	randomised, double-blind, parallel-group, comparator-controlled	E10 + A10/A10	15	15	−19.9	(−32.4,-7.4)	6.3	(−7.2,19.8)	−14.4	(−27.7,-1)	−33	(−54.1,-11.9)
Blagden,M.D., 2007,UK,25	randomised, double-blind, parallel-group, placebocontrolled	E10 + A10/A10	72	76	−14.1	(− 17.9,-10.2)	−0.3	(−4.3,3.6)	−9.2	(− 12.4,-6)	−8.3	NR
Bays,H.E., 2011,USA,26	multicenter, randomized, double-blind, parallel-group	(a)E10 + A20/A40 (b)E10 + A20/A40 (c)E10 + A40/A80 (d)E10 + A40/A80	73 19 176 101	64 28 159 120	−21.9 −13.8 − 15.4 − 18.3	(− 28.1,-15.6) (− 24.6,-3) (− 19.4,-11.3) (− 23.2,-13.3)	3 − 0.1 − 0.2 1.5	(−1.9,7.9) (− 8.7,8.5) (− 2.3,1.9) (−1.1,4.1)	− 13.2 − 9.4 − 9.6 − 10.7	(− 17.5,-9) (− 16.7,-2.1) (− 12.3,-7) (− 13.9,-7.4)	−7.6 − 11.8 − 6.7 − 7.9	(−19,3) (− 25.4,1.3) (− 12.5,-1.2) (− 14.5,-1.7)
Conard,S.E., 2008,USA,27	multicenter, randomized, double-blind, parallel-group	E10 + A20/A40	92	92	−20	(−25,-15)	2	(−2,7)	−12	(− 16,− 9)	-9	(− 18,0)
Conard,S.E., 2010,USA,28	multicentre, randomized, double-blind, parallel-group	(a)E10 + A40/A80 (b)E10 + A40/A80 (c)E10 + A40/A80	61 67 149	66 68 145	−14.3 − 16 − 17.4	(− 20.9,-7.8) (− 22.3,-9.6) (− 21.7,-13.1)	2 1.9 − 0.8	(−1.4,5.4) (−1.4,5.2) (− 3,1.4	−8.9 − 10.1 − 10.4	(−13.2,-4.6) (− 14.2,-5.9) (− 13.2,-7.6)	−6.4 − 7.2 − 7.7	(− 15.1,2.3) (− 15.9,1.6) (− 13.6,-1.8)
Leiter,L.A., 2008,Canada,29	multicenter, randomized, double-blind, parallel-group	E10 + A40/A80	277	279	−16	(−19,-13)	0	(−1,2)	−10	(− 12,-8)	− 7	(− 11,-3)

### Statistical analysis

Primary analyses assessed the continuous data about the changed LDL, HDL, TC and TG from baseline to endpoint between experimental and controlled groups. If the study only reported the data which included baseline values and endpoint values instead of the change values, we could use the Cochrane Handbook 16.1.3 to solve the missing data [12]. The Chi-squared test based Q-statistic and I^2^ statistics were was used to estimate the heterogeneity (I^2^ ≤ 25%, low heterogeneity; 25% < I^2^ < 50%, moderate heterogeneity; I^2^ ≥ 50%, high heterogeneity) [14]. A fixed-effects model was used to pool the results when heterogeneity was ≤50%, while a random-effects model when heterogeneity was > 50% was selected [15, 16]. Sensitive analysis was performed to investigate the influence of a single study on the overall pooled estimate by deleting one study in each turn. Publication bias was evaluated by the Begg’s and Egger’s test [17, 18](*p* < 0.05 was considered representative of statistically significant publication bias). Stata version 12.0 software was used for the meta-analysis.

## Results

### Description of the studies

**Fig. 1.**
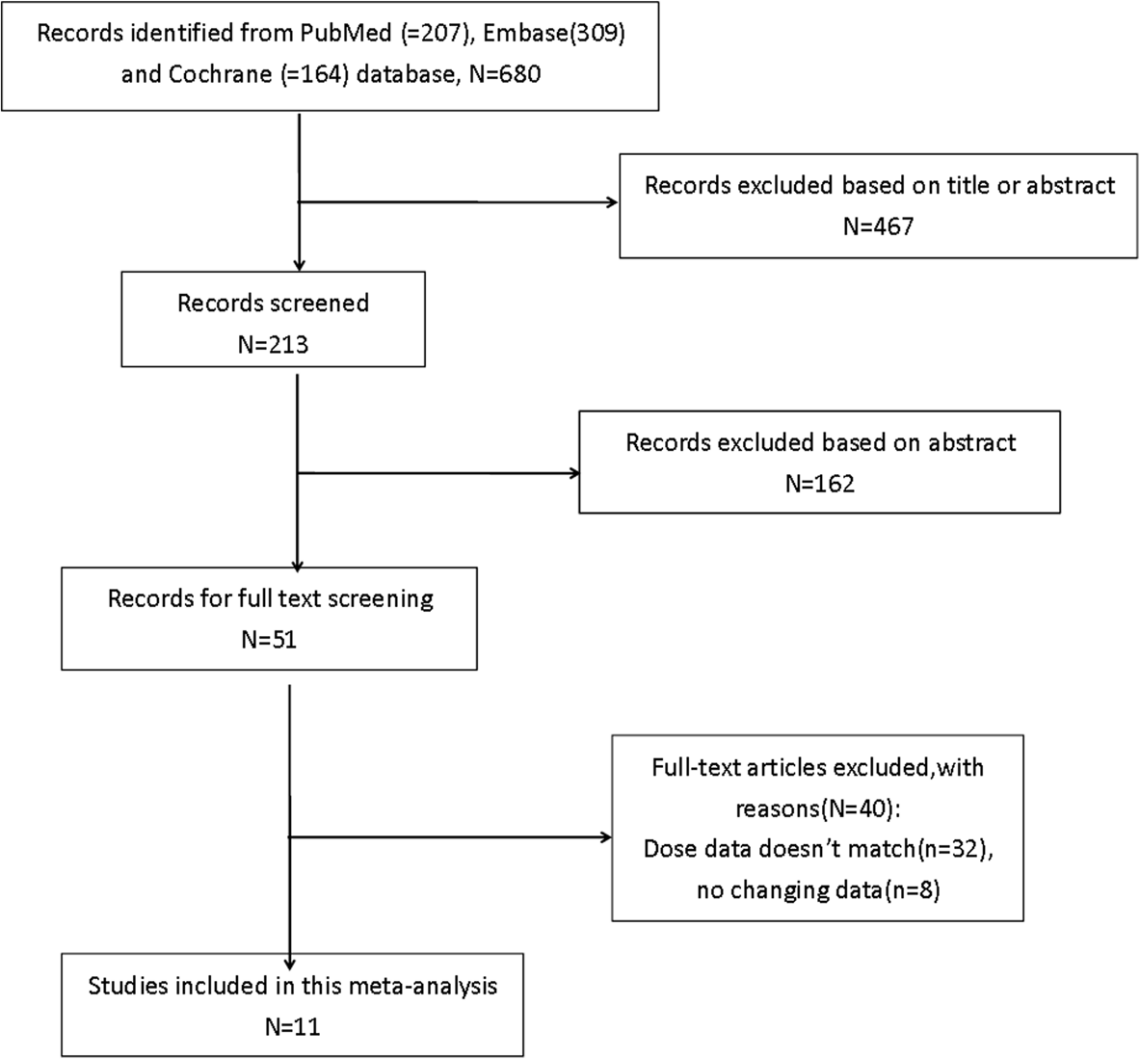
Eligibility of studies for inclusion in meta-analysis

**Fig. 2.**
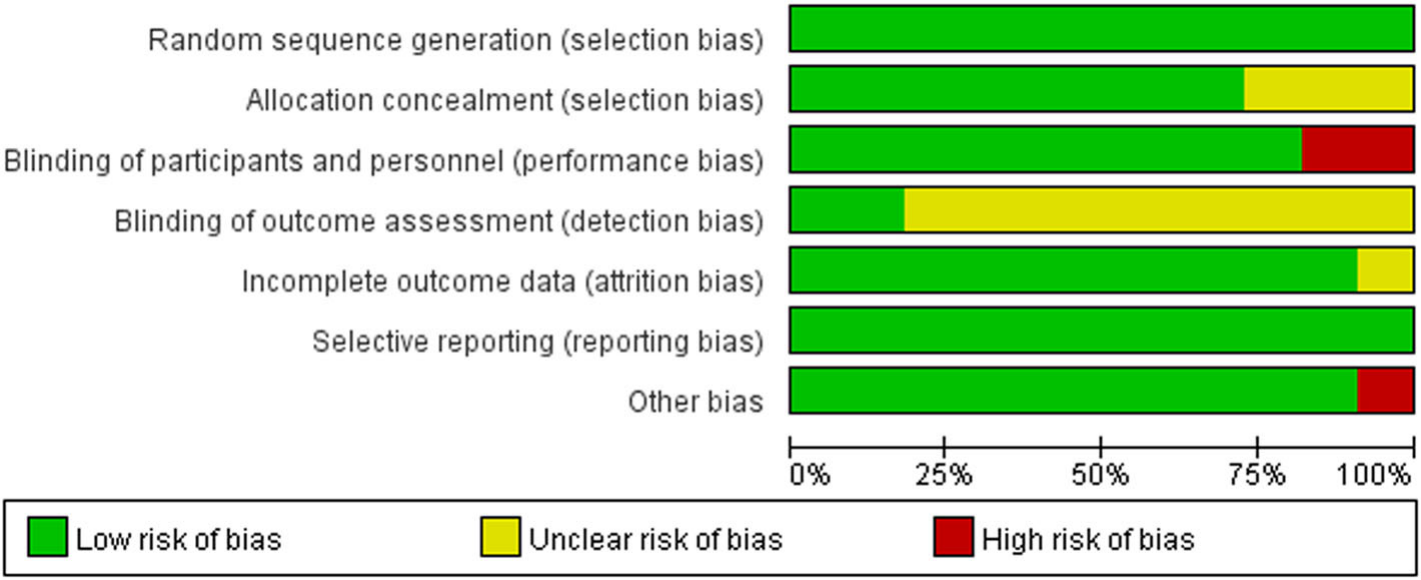
Risk of bias in the included studies

After primary search from the three databases, 680 studies were recruited (207 in Pubmed, 309 in EMbase, 164 in Cochrane). Then 467 studies were excluded by reviewing the title, and 162 ones were excluded by reviewing the abstract. Forty studies were left according to the exclusion and inclusion criteria (Fig. 1). Finally, 11 studies [19–29] with 5206 participants were included in this meta-analysis (Table 1). Three studies were from Asia, four from US, and four from Europe. All randomized controlled trials (RCTs) were carried out for more than 4 weeks. All trials were randomized, parallel-group studies and 9 trials were double-blind. The patients with LDL-C level > 70 mg/dL (at high risk of CHD) or with hypercholesterolaemia were included in the trials. All included studies were evaluated in terms of the risk of bias using the Cochrane risk of bias tool and the details were presented in Fig. 2.

### Low-density lipoprotein cholesterol (LDL-C)

**Fig. 3.**
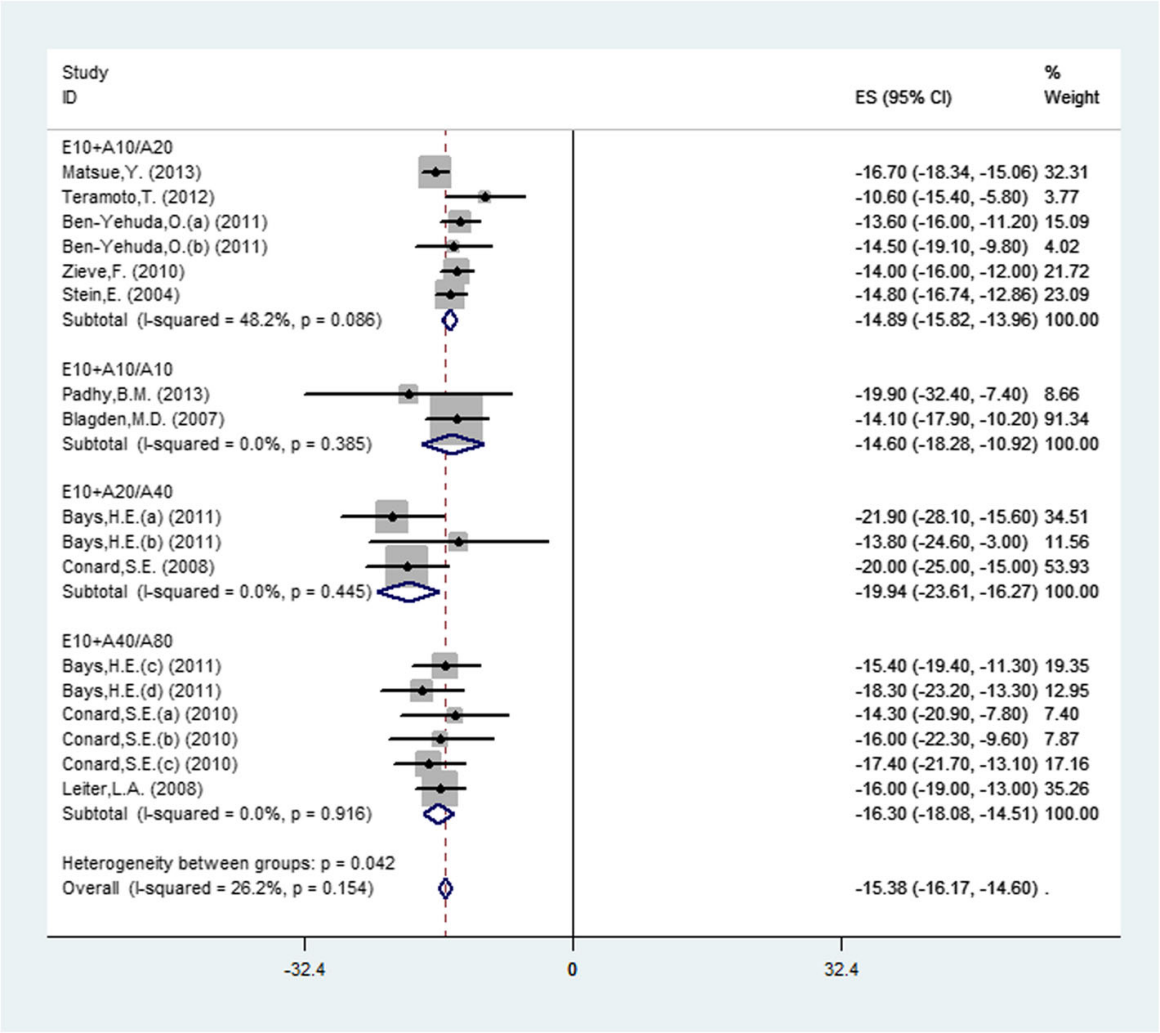
Forest plot showing the overall and subgroup analysis about the comparison between combination therapy and monotherapy in LDL-C according to different doses (E10+A10/A20, *P* < 0.0001; E10+A10/A10, *P* < 0.0001; E10+A20/A40, *P* < 0.0001; E10+A40/A80, *P* < 0.0001)

Seventeen studies (11 publications) investigated the change of LDL-C from baseline to endpoint between experimental and controlled groups. There were six studies (5 publications) investigating the combination therapy of Ezetimibe (10 mg) and Atorvastatin (10 mg) (E10 + A10) versus Atorvastatin (20 mg) monotherapy (A20), two studies (2 publications) reporting E10 + A10 vs. A10, three studies (2 publications) reporting E10 + A20 vs. A40 and six studies (3 publications) reporting E10 + A40 vs. A80. Pooled data using a fixed-effects model displayed that combination therapy led to a significant reduction in LDL-C (MD = − 15.38, 95% CI: -16.17 to − 14.60, *P* < 0.0001) with moderate heterogeneity (*P* = 0.12, I^2^ = 26.2%) among studies (Fig. 3). The results showed that the four doses were significant and the E10 + A20 vs. A40 group was the most obvious (MD = − 19.94, 95% CI: -23.61 to − 16.27, *P* < 0.0001), by subgroup.

### High-density lipoprotein cholesterol (HDL-C)

**Fig. 4.**
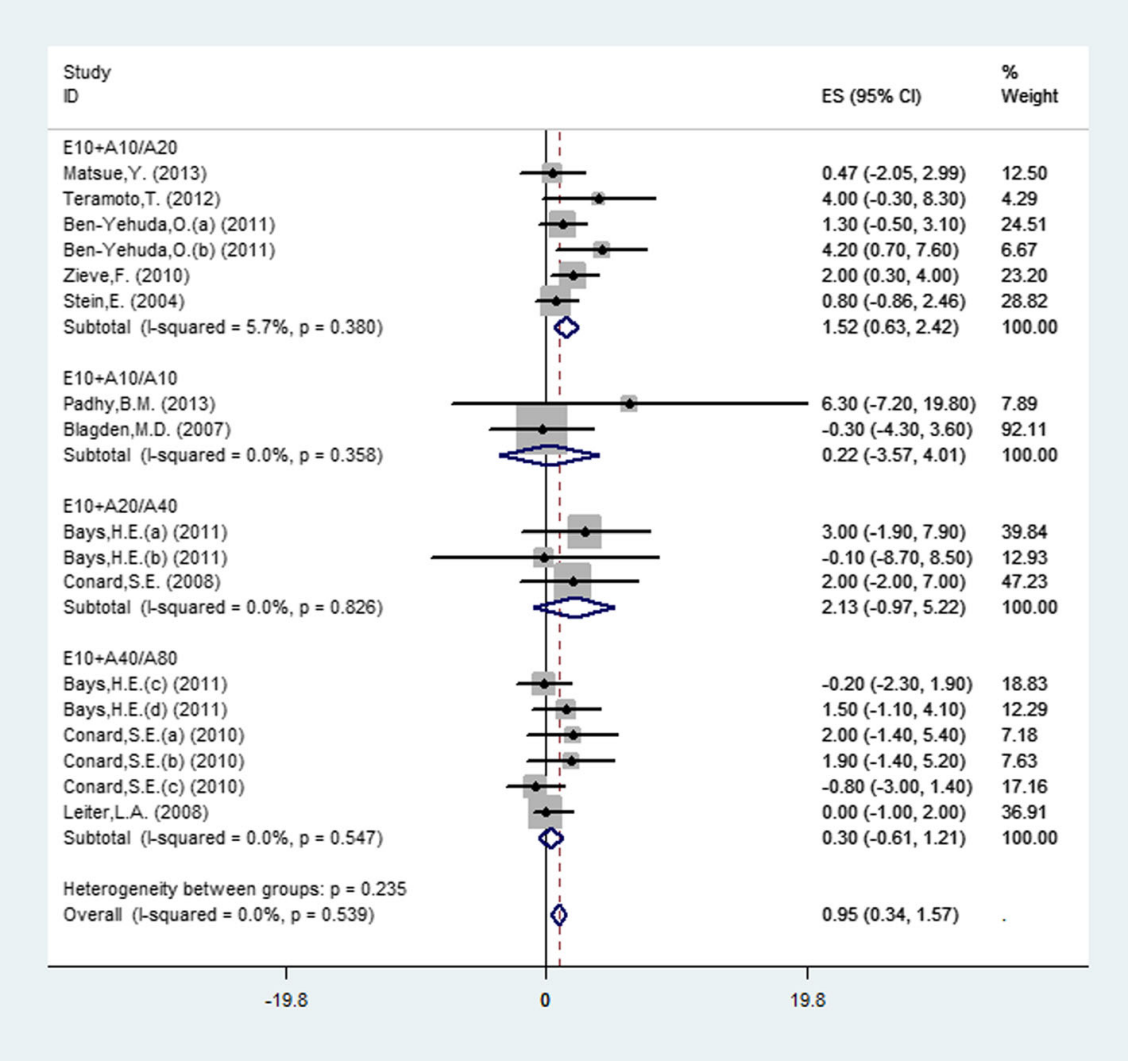
Forest plot showing the overall and the subgroup analysis about the comparison between combination therapy and monotherapy in HDL-C according to different doses (E10+A10/A20, *P* = 0.001; E10+A10/A10, *P* = 0.909; E10+A20/A40, *P* = 0.178; E10+A40/A80, *P* = 0.522)

Seventeen studies (11 publications) investigated the change of HDL-C from baseline to endpoint between experimental and controlled groups (Fig. 4). Pooled estimates using a fixed-effects model displayed that, no heterogeneity existed among studies (*P* = 0.539, I^2^ = 0%). The results showed that the overall efficacy was significant difference between combination and monotherapy (MD = 0.95, 95% CI: 0.34 to 1.57, *P* = 0.002) and the E10 + A10 vs. A20 group was the most obvious (MD = 1.58, 95% CI: 0.72 to 2.44, *P* = 0.0003), by subgroup.

### Total cholesterol (TC)

**Fig. 5.**
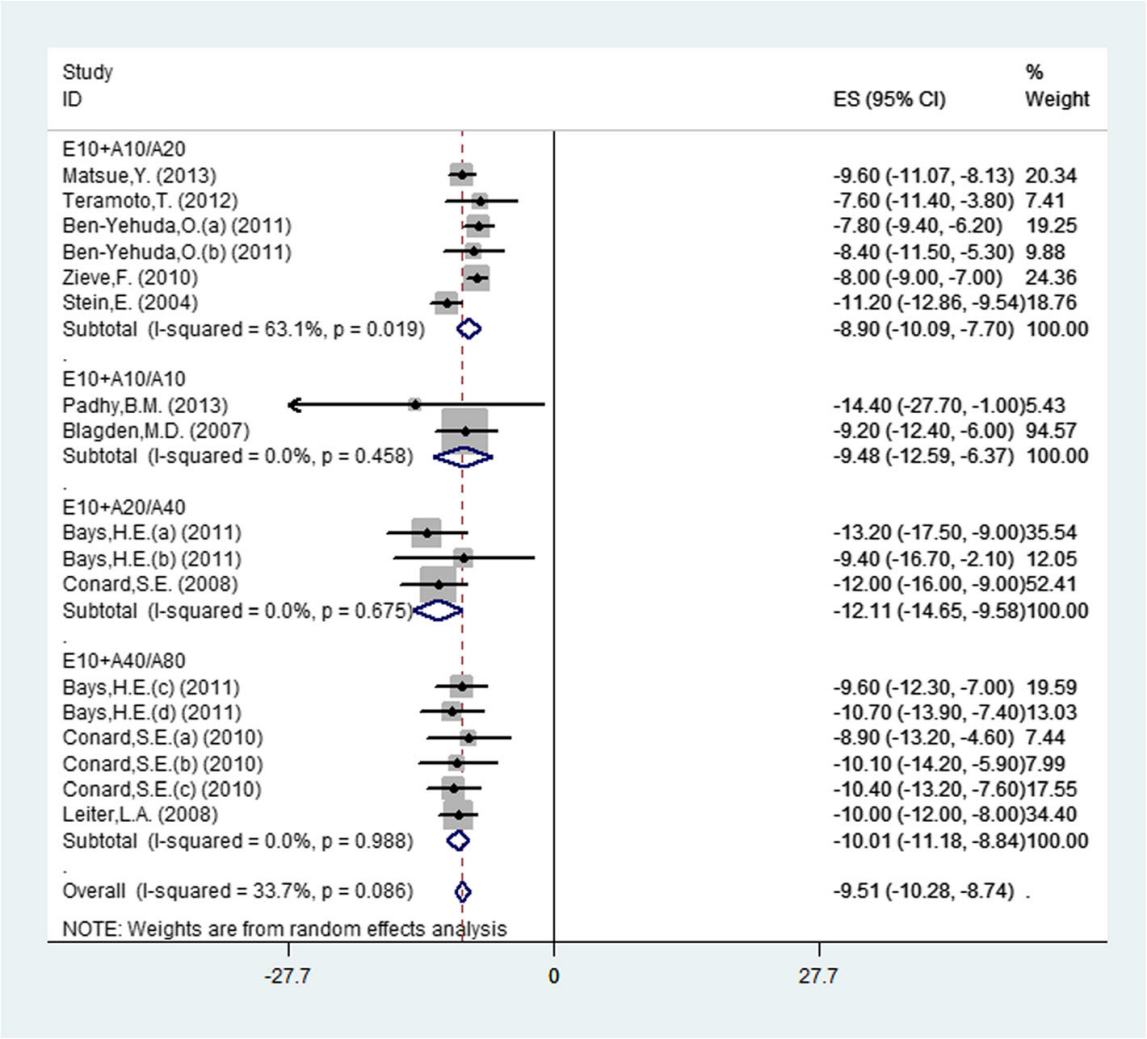
Forest plot showing the overall and the subgroup analysis about the comparison between combination therapy and monotherapy in TC according to different doses (E10+A10/A20, *P* < 0.0001; E10+A10/A10, *P* < 0.0001; E10+A20/A40, *P* < 0.0001; E10+A40/A80, *P* < 0.0001)

Seventeen studies (11 publications) reported the TC changes and random-effects model was used to analyze the outcome because of the moderate heterogeneity among studies (*P* = 0.086, I^2^ = 33.7%). There was significant difference between combination and monotherapy (MD = − 9.51, 95% CI: -10.28 to − 8.74, *P* < 0.0001). The results showed that there was significant difference in the four doses (Fig. 5) and the E10 + A20 vs. A40 group was the most obvious (MD = − 12.11, 95% CI: -14.65 to − 9.58, *P* < 0.0001), by subgroup.

### Triglyceride (TG)

**Fig. 6.**
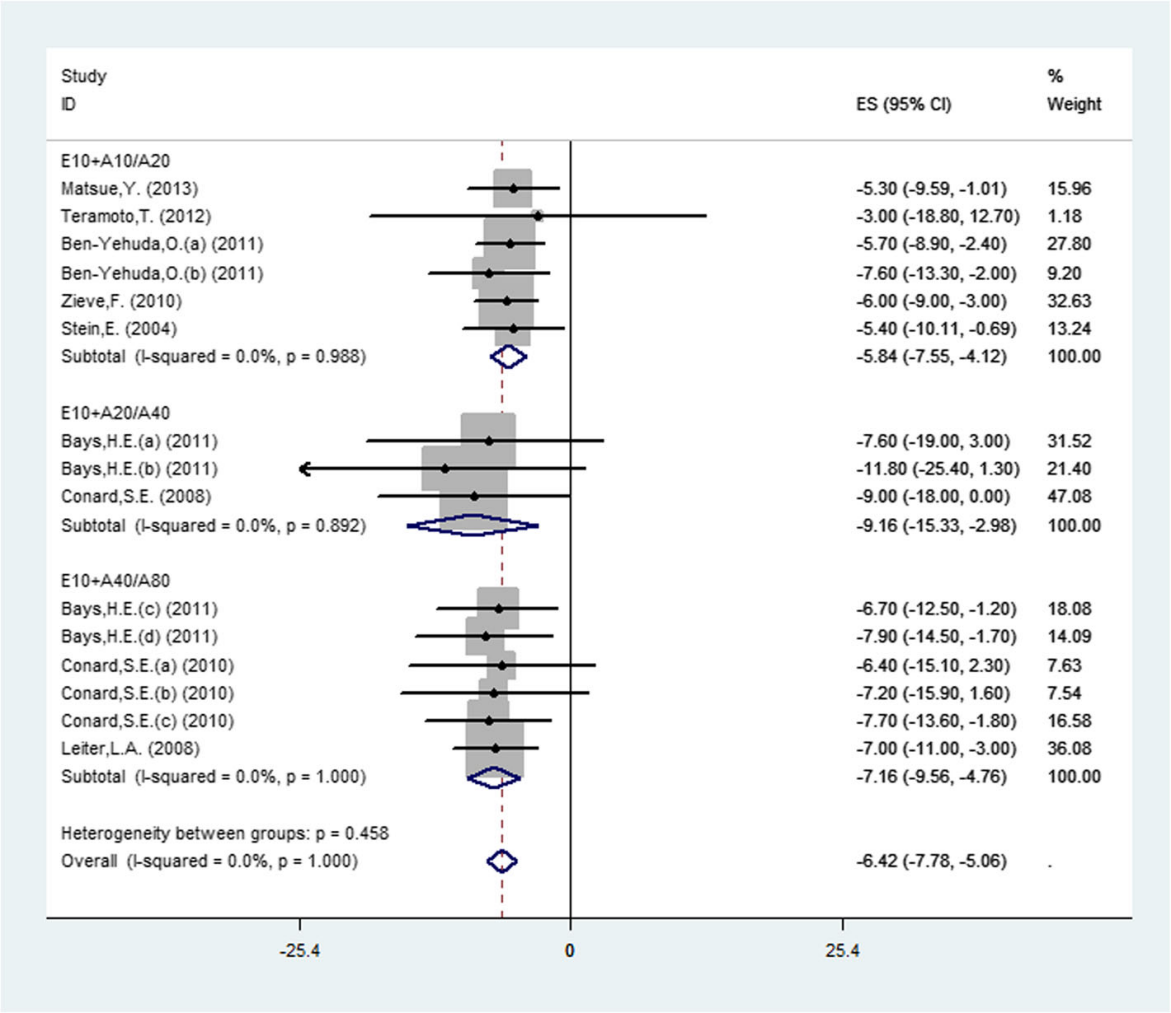
Forest plot showing the overall and the subgroup analysis about the comparison between combination therapy and monotherapy in TG according to different doses (E10+A10/A20, *P* < 0.0001; E10+A20/A40, *P* = 0.004; E10+A40/A80, *P* < 0.0001)

Fifteen studies (9 publications) reported the TG changes and pooled data using a fixed-effects model displayed that combination therapy led to a significant reduction in TG (MD = − 6.42, 95% CI: -7.78 to − 5.06, *P* < 0.0001) with no heterogeneity (*P* = 1.00, I^2^ = 0%) among studies (Fig. 6). Due to the E10 + A10 vs. A10 group only including one study, three doses were included in the forest plot. The results showed that there was significant difference in the three doses and the E10 + A20 vs. A40 group was the most obvious (MD = − 9.16, 95% CI: -15.33 to − 2.98, *P* = 0.002), by subgroup.

### Sensitivity analysis

Sensitivity analysis was implemented to evaluate the results and we found that all of the results remained relatively stable by excluding individual studies.

### Publication bias

In the LDL dose group, the results showed that no significant bias existed in the pooled data by the Begg’s (Z = 1.44, *P* = 0.149) and Egger’s test (*t* = − 0.30, *P* = 0.768).

## Discussion

The results of this meta-analysis showed that the overall effectiveness of combination therapy of Ezetimibe and Atorvastatin was significantly better than Atorvastatin monotherapy on lowering LDL-C, TC and TG among all the four doses comparison (E10 + A10/A20; E10 + A10/A10; E10 + A20/A40; E10 + A40/A80). Besides, we also found a significant effect on raising HDL-C which was different with previous individual studies. Sensitivity analysis showed that the results remained relatively stable by excluding individual studies.

Although there were some studies reporting that coadministration of Ezetimibe with a statin was more effective than statin monotherapy in lipid-lowering, the reports about Ezetimibe-Atorvastatin and doubling Atorvastatin dose monotherapy were limited. One published qualitative systematic review without quantitative analysis showed that the combination therapy of Ezetimibe and Atorvastatin should facilitate reaching therapeutic goals in terms of LDL cholesterol among patients with severe hypercholesterolaemia. And it was written by French [10]. In another meta-analysis carried out by Chinese [30], the doses were varied in included trials. Only LDL-C was discussed, and the conclusion was similar to mine. Hence, to the best of our knowledge, this study is the first meta-analysis to compare the effects of combination therapy of Ezetimibe and Atorvastatin with Atorvastatin monotherapy about four doses. What’s more, this study involved the comparison of four dose groups commonly used in clinic, so that the results may be more intuitively in selecting drugs dose.

Ezetimibe is known as cholesterol absorption inhibitor, selectively inhibiting the absorption of cholesterol from the intestinal lumen into enterocytes, different from other types of lipid-lowering drugs on mechanism of action. Ezetimibe inhibits cholesterol absorption through external sources and is administered in conjunction with statins which inhibits cholesterol synthesis through internal sources. The combination therapy can complement the regulation of lipid levels and can get better effect in lipid-lowing [5, 31, 32]. Though the statins are generally safe in long-term treatment, there are still adverse reactions or potential risks especially when the dosage was doubled or even tripled. The statins adverse effects are dose dependent, and risk is amplified by drug interactions [33]. For participants, simply raising dose of statin for lipid-lowing can increase the risk. Besides, a study has suggested that Atorvastatin and Ezetimibe have no relevant pharmacokinetic drug–drug interaction [34]. So, we should find some ways to solve this problem.

### Limitations

Some limitations have to be mentioned here: (1)In the searching literature, several conference papers were unable to obtain the full text and were not included in the study [35–38]. Furthermore, four papers [39–42] can’t be included,in which the existing data was not enough to calculate the standard deviation (SD). (2) Only four publications [21, 22, 27, 29] provided the incidence of adverse events about ALT, AST, CK or GI. As a result, no analysis about security was performed. Owing to unclear type of long-term security, more clinical trials with high quality, large samples and long-term following-up were needed to identify the security of such interventions. (4)In Yuya Matsue’s [19] report, it provided the values from baseline and endpoint without SD. The missing data was calculated in the method of the Cochrane Handbook 16.1.3 [12], which might affect the final results of the meta-analysis.Due to the inclusion of a small quantity of literature, more clinical trials with high quality, large samples and long-term following-up are needed to support the results. And more useful data are required in the literature, such as MD, ME or 95% confidence interval which will be more convenient for clinicians to master the data and also will facilitate epidemiologists to analyse big data.

## Conclusion

The overall efficacy and subgroup’s efficacy of combination therapy of Ezetimibe and Atorvastatin on lowering LDL-C, TC and TG was significantly better than Atorvastatin monotherapy’s. The overall and the E10 + A10/A20 group’s effectiveness of combination therapy on rasing HDL-C were significantly.

## Abbreviations

A10: Atorvastatin (10 mg) monotherapy
A20: Atorvastatin (20 mg) monotherapy
A40: Atorvastatin (40 mg) monotherapy
A80: Atorvastatin (80 mg) monotherapy
CHD: Coronary Heart Disease
E10 + A10: Ezetimibe (10 mg) and Atorvastatin(10 mg)
E10 + A20: Ezetimibe (10 mg) and Atorvastatin (20 mg)
E10 + A40: Ezetimibe (10 mg) and Atorvastatin (40 mg)
HDL-C: High-density lipoprotein cholesterol
LDL-C: Low-density lipoprotein cholesterol
RCTs: Randomized controlled trials
TC: Total Cholesterol
TG: Triglyceride
